# Predictive Factors for Severe Maternal Morbidity in Brazil: A Case-Control Study

**DOI:** 10.3390/healthcare9030335

**Published:** 2021-03-16

**Authors:** Daniela Mendes dos Santos Magalhães, João Marcos Bernardes, Carlos Ruiz-Frutos, Juan Gómez-Salgado, Iracema de Mattos Paranhos Calderon, Adriano Dias

**Affiliations:** 1 Gynecology, Obstetrics and Mastology Posgraduate Programme, Botucatu Medical School, São Paulo State University (UNESP), Botucatu, Sao Paulo 18618-687, Brazil; danymmagalhaes81@gmail.com (D.M.d.S.M.); iracema.calderon@gmail.com (I.d.M.P.C.); 2 Secretary of State for Health of the Federal District, Brasilia 70390-150, Brazil; 3 Public (Collective) Health Grade Programme, Botucatu Medical School, São Paulo State University (UNESP), Botucatu, Sao Paulo 18618-687, Brazil; jmbernardes@yahoo.com (J.M.B.); dias.adriano@unesp.br (A.D.); 4 Department of Sociology, Social Work and Public Health, Faculty of Labour Sciences, University of Huelva, 21007 Huelva, Spain; frutos@uhu.es; 5 Safety and Health Posgraduate Programme, Universidad Espíritu Santo, Samborondón, Guayaquil 092301, Ecuador

**Keywords:** women health, maternity, severe maternal morbidity, near miss, health systems, quality of care, healthcare, Millennium Development Goals

## Abstract

The maternal mortality or "maternal near miss" ratio in Brazil reflects the socioeconomic indicators as well as the healthcare quality in some areas of this country, pointing out fragile points in the health services. The aim of this study was to estimate the association of diverse variables related to pregnancy and the occurrence of Near Miss in a population of women who were cared in public maternity wards in Brazil. A case-control study was performed. The association between variables and outcomes was verified through a chi-square test. A multiple analysis was carried out, producing odds ratio (OR) estimates with values of *p*≤0.25 in the univariate model. The results point to the following risk factors for Severe Maternal Morbidity: non-white (<0.001, OR 2.973), family income of up to two minimum wage salaries (<0.001; OR 2.159), not having a partner (<0.001, OR 2.694), obesity (<0.001, OR 20.852), not having received pre-natal care (<0.001, OR 2.843), going to less than six prenatal appointments (<0.001, OR 3.498), undergoing an inter-hospital transfer (<0.001, OR 24.655), and the absence of labor during admission (<0.001, OR 25.205). Although the results vary, the incidence of women with potential life-threatening complications is high in Brazil, which reinforces the need to universalize more complex interventions as well as coverage of primary care. The presence of precarious socio-economic indicators and unqualified obstetric care were risk factors for Severe Maternal Morbidity.

## 1. Introduction

The Millennium Development Goals [1] established the reduction of maternal mortality and the enhancement of maternal health amongst main objectives. Maternal deaths are a rare event, but their occurrence transcends individual and family spheres. In low-middle income countries, they are determined by socioeconomic conditions and obstacles to care, making them an important indicator of a given population’s social reality, revealing situations of health and gender inequality which are inversely proportional to human development levels. However, maternal mortality, despite being a suggestive and sensible indicator, is not by itself capable of reflecting the quality of obstetric care [2,3,4].

In addition to specific studies about maternal mortality, since the 1990s there have been studies about Severe Maternal Morbidity (SMM). These were based on a persistent concern with the quality of obstetric care offered to women in the pregnancy/childbirth cycle, but the definition of SMM remains imprecise. While maternal deaths are considered a single, clear event, SMM is a continuum which starts with the occurrence of a complication during pregnancy, childbirth, and puerperium. Possible outcomes are a positive resolution or death. In other words, there’s no precise manner of determining at which point maternal morbidity becomes severe or extremely severe [5].

Currently, SMM is defined as happening when a pregnant woman has a Potentially Life-Threatening Condition (PLTC), subdivided into Near Misses (NM), in which women are effectively under risk of dying through organ failure but survives, and non-near miss maternal morbidity, which includes all other lower-risk PLTCs [6]. NM is defined as a situation where woman who almost died but survived complications during pregnancy, childbirth, or up to 42 days after childbirth [7]. To be classified as a NM, a woman should present at least one clinical, laboratorial, or management criteria among the parameters established by the WHO since 2009 [6,7].

The study of NM arose as an alternative to some obstacles facing studies about maternal mortality, such as the low total number of cases and underreporting. It is known that for each woman who dies from pregnancy, childbirth, and puerperium complications (be they hemorrhagic, hypertensive, or infectious), at least 20 survive, and their complications are analogous to those of women who died [8]. Beyond enabling larger sample groups, the study of survivors enhances the robustness of analyses and enables the collection of victim’s accounts, an important tool to evaluate every level of obstetric care and to enhance assistance to women [6,8].

Thus, this study has the objective of estimating the association of diverse variables which may or may not be direct related to pregnancy and the occurrence of NM in a population of women who were cared for in public maternity wards in Brazil’s Federal District.

Since Brazil is a highly unequal low-middle income country, whose public health system is unable to consistently provide adequate care to pregnant women due to financial and organizational constraints, our hypothesis was that the associated factors would be related to sociodemographic factors and to the quality of care provided to these women.

## 2. Materials and Methods

### 2.1. Design

The present study is a hospital-based case-control study with primary data, whose sample group included women in pregnancy, childbirth, or puerperium who were hospitalized due to PLTCs. 

### 2.2. Setting

The Federal District is one of the 27 federative units of Brazil, encompassing the capital, Brasilia, and 31 more territories known as Administrative Regions (RAs). The nine hospitals included in this study are responsible for obstetric care in 29 RAs, therefore accurately representing regular and high-risk obstetric care scenarios for obstetric care in the public health system of Federal District.

The development of this work complied with all the ethical principles set out in the Helsinki Declaration. Participants were previously informed of the purpose and procedure of the study and gave their written consent to voluntarily participate. Subjects involved in the study were not exposed to any risk. This study was carried out in nine public hospitals under the State Secretariat of Health of the Federal District, and it was approved by the Research Ethics Committee of the Health Sciences Research and Education Foundation.

### 2.3. Participants

The minimum sample size was estimated as 532 women, distributed in 174 cases and 348 controls, considering an estimate of 20 NMs per 1000 live births (LB) and 50 maternal deaths for per 100.000 LB [9], assuming 5% as the lowest proportion of exposition between controls, corrected for type I (5%) and type II (20%) error effects, respectively.

The cases were women in pregnancy, childbirth, and puerperium who were hospitalized in the selected hospitals and presented criteria for PLTC (women who evolved to conditions of severity with no organ failure) or NM (who evolved to conditions of severity with organ failure, independently of gestational age), following the criteria proposed by the WHO [6]. Two controls were selected for each case. These were women who were hospitalized during the same period in the selected service providers, with a similar gestational age to a case (± one week) but with no PLTC or NM criteria. Five women who died after being interviewed and classified into PLTC or NM were not included, as well as cases who reached the services dead and women who died before being interviewed and classified.

### 2.4. Instrument

The data, obtained in single structured interviews during hospitalization, was recorded in an instrument designed specifically for this study, based on the Questionnaire on SMM [10] which, beyond classifying cases into PLTC or NM, included questions relating to sociodemographic conditions and clinical-obstetric profile. The demographic data were self-reported and BMI data were collected from medical records. The questionnaire on SMM was validated in the Brazilian study “Development and validation of a questionnaire to identify severe maternal morbidity in epidemiological surveys” [10]. The main researcher was trained to apply the questionnaire and was responsible for all data collection.

### 2.5. Data Analysis

All statistical analyses were done using the IBM/SPSS v.25.0 software (IBM Corp., Armonk, NY, USA). Firstly, the distribution of the different set of independent variables (sociodemographic, clinical profile, and obstetric care) was verified for both PLTC and NM cases. Despite some suggestion in the literature that PLTC and NM are not distinct from each other, the differences were tested by way of percentage distribution (chi-square tests), defining the case (PLTC + NM) and control groups.

Next, independent associations between each predictive variable and outcome (case or control) were tested by logistic regression model. Among the different studies that have been undertaken in the investigation of risk factors for NM, the most common data analysis methods are multiple logistic regression models, in which predictive variables are treated as if they were situated in a single hierarchical level of determination, without considering the different levels of explanation that determine morbidity conditions [11,12]. Despite being the most traditional model, its characteristics did not seem to be the best suited to analyze the collected information, and it was decided to adjust the multiple logistic regressions model by three previously defined hierarchical blocks composed by those variables that had produced odds ratio estimates (OR) with values of *p* ≤ 0.25 in the simple logistic model [13]. This method of hierarchical analysis was chosen because it is capable of testing hypotheses built from concepts from distal to proximal determination to the studied phenomenon [14], which was SMM in our investigation. Each variable block’s hierarchical level was considered in the chain of social determination of outcomes, assuming distal factors influence proximal factors. The distal predictive level was constituted by socioeconomic factors like age, level of education, family income, perceived ethnicity (white and non-white—participants that self-identified as Caucasian were categorized as white, while non-white primarily consisted of participants that self-identified as black and as mixed ethnic ancestries, known as “pardos” in Brazil. It also included east Asian and indigenous; however, these represented less than 1% of the participants), and relationship status; the proximal level was formed by obstetric care factors like prenatal in the public system (Brazil’s unified health system), prenatal in area of coverage, previous c-sections and abortion, parity, frequency of prenatal care, method of service access, onset of labor; and the intermediary level by clinical profile like alcohol and tobacco use, personal risk antecedents, BMI classification, and regular exercise practice. 

To adjust the multiple logistic regression model, variables were included into blocks, from the distal category (socioeconomic variables) to the intermediary (clinical profile) and proximal (obstetric care) categories. Starting from the second block, variables were kept in the model if they were still associated (*p* ≤ 0.25) after an adjustment to the previous block. After the inclusion of the last block, variables that had lost statistical significance were removed from the final model, following an order from largest to smallest *p*-value. Only variables with risk factors of *p* ≤ 0.25 were kept. Multiple adjustments were carried out through a stepwise forward selection method.

## 3. Results

During the research period, 179 SMM cases were identified. Five women passed away after the interview and were not included in the final sample, bringing the total number of women who participated in the study as cases to 174. They were classified as SMM according to the research criteria. The final ratio of cases-controls was of 1:2.06, since 358 control were selected. The main causes of maternal morbidity in the sample group were hypertensive (50.6%), infectious (33.9%), and hemorrhagic (33.3%) conditions.

The global average age of interviewees was 26.9 years old (± 6.8), with a median age of 27 years old. Among cases, average age was 28.02 (±7.1), with a median of 28, while among controls it was 26.3 (±6.6) and a median age of 26, differences which were not statistically significant. 

Table 1 shows the distribution of the following independent variables for both NM and PLTC: age, education level, relationship status, family income, perceived ethnicity, prenatal care, alcohol and tobacco use, personal risk antecedents, and previous abortions and c-sections. Following predictions from planning and from the literature [3], no statistically meaningful differences were found between PLTC and NM, and both categories were grouped together as cases. Hospital transfers were made in situations where the women’s need for care was greater than the health unit´s ability to meet these demands. In Brazil, the transfer of a pregnant woman to a tertiary referral hospital must be done with the assistance of a regulatory center belonging to the system. This system daily checks the number of beds available in these units and decides, according to the geographic location and available resources, where to transfer the specific case.

Table 2 presents the distribution of variables between cases and controls. Statistically significant differences arose between these groups in socioeconomic, clinical profile, and obstetric care characteristics such as age (0.030), relationship status (<0.001), family income (<0.001), perceived ethnicity (<0.001), regular exercise practice (<0.001), preexisting clinical conditions (0.003), previous c-sections (0.023), previous abortions (0.024), BMI classification (0.001), prenatal condition with area of coverage (<0.001), trimester of start (<0.001), number of appointments (<0.001), and cause of labor onset (<0.001).

Table 3 shows the results of the univariate logistic regression models and Table 4 presents the results of the hierarchical multiple logistic regression model. Differences between cases and controls are statistically significant in the multiple models in relation to be non-white (OR 2.9; IC 1.9-4.4; *p*-value < 0.001), not having a spouse (OR 2.6; IC 1.7–4.1; *p*-value < 0.001) and family income of up to two minimum wages (OR 2.1; IC 1.4–3.1; *p*-value < 0.001). These predictive factors elevate the risk of severe outcomes by two to three times.

In relation to clinical profile, some previous clinical conditions, like subarachnoid hemorrhage (SAH), falciform anemia and heart, kidney and neurological diseases are statistically significant protective factors. Being obese or overweight, as well as not exercising, are risk factors for SMM.

In the obstetric care block, not having pre-natal care in area of coverage entails a twofold elevation in the chance for an adverse outcome, while inter-hospital transfers greatly elevate this risk. Having less than six pre-natal appointments or not having them altogether also leads to a significant increase in the chance of a severe outcome.

## 4. Discussion

Discussions on maternal mortality and morbidity studies reflect, in general, inequities to which women are exposed, such as socio-demographic, economic, gender, race, or access to health services.

Hierarchical modeling is applicable to epidemiologic studies with a great number of predictive factors and in situations in which one can establish conceptual frameworks of intrinsic relations between variables in the same set, which would, theoretically, be independent of the other sets, kept in their groups during data analysis. Therefore, beyond a quantitative approach which preserves some qualitative issues, this decision can reduce the number of terms needed to test associations as well as the risk of saturation by excess of variables. Analyses developed in this logic have the advantage of being able to incorporate effects and interactions of factors relating to life conditions, social, and economic structures and context, to issues regarding access to health services and others connected to physiological issues and health-related behaviors [14].

In studies in which the unit of observation is the individual, the use of analysis models in which variables are organized in distinct hierarchical levels and oriented by a corresponding theorical model allows for the interpretation of results to go beyond simply detecting the presence or absence of statistical association [14]. This seeks to reduce distortions in the estimation of the effects of the distal (socioeconomic) determinants, enabling a wider interpretation of the health situation of this population.

In this study, hierarchical analysis allowed for the identification of characteristics which, independently of age, education level and family income elevate the risk of adverse outcomes. These conditions are not directly responsible for the occurrence of SMM, but they favor the proximity of certain determinants and reinforce gender inequalities: Because they are conditions which impose difficulties in access to health care, education and income are limiters of women’s capacity to protect their own health [15].

Low income and being non-white are both associated to adverse outcomes in the univariate analyses. This association was not mitigated with the introduction of the obstetric care variables and was still statistically significant in the final model. Waterstone et al. [16] found independent association between social exclusion (characterized by being under 16 years old and having low income and/or inadequate housing) and NMs, in a similar way to the association between markers related to social exclusion (income and black woman) found in this study.

It is known that being a black woman is a risk factor in obstetric care and delivery outcomes. Studies have shown that black women experience high rates of pregnancy-related mortality and morbidity, along with higher rates of cesarean delivery, as compared with other racial and ethnic groups. When compared to white women, black women are more likely to have adverse outcomes [17].

Access to and effectiveness of health actions are strongly influenced by socioeconomic indicators like education level and family income. In obstetric care, the most frequent differentials oftentimes reproduce social inequalities, as does the provided assistance [18]. Similarly, to some of this study’s results, other studies have already demonstrated a larger incidence of NM in non-white, older women with little education and low income [19,20,21].

Women who live with no spouse tend to be more vulnerable and present a higher chance of having had inadequate prenatal care—be it due to less motivation to use health services; to stigmatization; or to lack of emotional, social, and affective support and the incentive to care for oneself—becoming more exposed to complications which may lead to NM cases [22,23,24]. This is corroborated by this study’s findings, which have shown that the risk of SMM is twice as high in women who declare they do not live with partners.

The presence of biological risk factors, such as a sedentary lifestyle and pre-existing clinical conditions also affects the outcome. Conditions like SAH, neurological, heart, and kidney diseases and falciform anemia behave as protective factors against an adverse outcome, pointing to a need to care attentively to habitual risk cases: Women who presented pre-existing morbidities demand greater attention and care and end up, apparently, having closer-monitored pregnancies. NM predictors can be categorized into three groups: those which are changeable, such as barriers to access, availability and usage of adequate health equipment according to each woman’s needs; non-changeable such as being a black woman; and finally, clinical factors which are sensitive to obstetric care [15]. The findings of this study reveal inequalities in access to care, since pregnant users who are identified as being high-risk benefit from additional care, while habitual risk women may end up being neglected and evolving to severe conditions which could be avoided through equal access.

The literature highlights that the provision of inadequate health services, intensified by the absence of the quality-protecting effect of opportunely provided health care, determines a higher frequency and severity of SMM [3,25,26,27].

An earlier start in prenatal care is a guarantee of qualified care and should not be limited to a set number of appointments. In the current study, Brazil´s unified health system was responsible for the prenatal care of most of the sample group, with a small proportion of women who did not go to any appointments. Not getting prenatal care exclusively in the National Health System and/or with no early start were risk factors for SMM, demonstrating that health coverage is a considerable aspect of providing care in the pregnancy/puerperium cycle, contributing to decisions about seeking health care services in case of complications and influencing the level of received care [27]. Generally, women in unfavorable socioeconomic conditions have a higher need to seek out free services offered by the Brazil’s unified health system, which corroborates this study’s findings. Even then, the results of this research revealed satisfactory prenatal care, with most cases starting care in the first trimester of pregnancy.

In relation to the number of appointments, having less than six was associated to adverse outcomes independently of the gestational trimester. This finding points towards a need for greater attention in identifying risk factors in women, and consequently, the monitoring, treatment, and opportune referrals of complications.

In Brazil, where most prenatal services are available to all pregnant women, few services are considered adequate, because, oftentimes, care is conditioned to complaints, and holistic health promotion actions are non-existent, hindering detection of complications in the pregnancy-puerperium cycle [28].

Prenatal care was near-universal in this study. There was, however, a high incidence of NM in the women who did not have it, corresponding to a 22-fold increase in the likelihood of SMM. The absence of prenatal care is a significant condition and strongly associated to an increased risk of adverse outcomes, as demonstrated in other studies, one of which concluded that 30% of women identified as NM had not had prenatal care and another in which the absence of prenatal care represented an eightfold increase in likelihood of a NM [25].

In the present study, most women spontaneously sought the reference hospital during prenatal care. However, not all of them were able to have the birth in the indicated service. A significant share of those who sought assistance in referenced services evolved to a condition of some gravity, having to be removed to another unit to access adequate support. This led to a higher incidence of cases and an expressive increment in the development of NM, as they demanded (programmed or unprogrammed) inter-hospital transfers to other services.

Pregnant women in labor are considered priority emergency cases [2]. The absence of adequate support for pregnant women in labor constitutes a health risk, as reinforced by the findings of this study, since being admitted before having the first signs of labor elevates the risk of SMM by 25 times. Therefore, the fragility or inexistence of a connection between prenatal-providing services and those who deal with delivery must be reviewed to enhance the efficiency of an organized system, be it through referrals and counter-referrals or care networks. However, a share of women in labor needed to transfer to other hospitals before they got adequate support, and even with a formal referral to the reference hospital, there was a delay in the realization of the prenatal/childbirth binomial.

Delay in obstetric care and in provision of adequate care resulted in severe maternal outcomes. Deeply understanding these delays may improve the quality of care, since it impacts decision making [23,25,29,30,31].

This study did not evaluate issues related to time or delays in actions. It did, however, identify a 24-fold increase in risk of a NM for women who used transfer services to access maternity wards with data related to the identification and arrival in an adequate health service (which can occur due to inadequacy or high costs in transportation infrastructure) [8].

This study revealed a threefold increase in SMM risk for deliveries with no labor, suggesting a risk increment in surgical interruption of pregnancy in women with no previous c-sections. Many studies have already demonstrated that surgical interruption of pregnancy is a NM risk factor [32,33].

Finally, it is important to discuss why WHO criteria for NM identification were chosen, since this choice can interfere with the ratio of NM. Until 2011, there was no standard criteria for identification of NM in the literature. It was only in 2010 that the WHO established a set of clinical, laboratorial, and management criteria for NM identification in an attempt to standardize them for different contexts14, differently from the criteria established by Mantel [33], Waterstone [16], or Geller’s [34] score system.

The WHO criteria are more specific, since they use a wide variety of markers based on the patients’ organic disfunction and management and, therefore, they identify more severe cases which could possibly lead to maternal death. In this way, the identification of NM cases among women with organic disfunctions enables the observation of women in extremely severe situations, which in turn allows us to establish better care standards for these situations [35].

However, it is not enough to identify NM cases if it is not possible to prospectively recognize potentially life-threatening conditions from which NM cases may arise. The similarities between NM and PLTC found in this study are characterized not only by biological aspects, but by other conditions of inequality in which these women find themselves, including race: white/non-white, education and family income. It is important to highlight that social vulnerability is reflected on access to health services, resulting in delayed pre-natal care, which interferes with the bond established between women and health care services during the period of pregnancy and puerperium. In both developed and low-middle income countries, gender, race, social class, and place of birth are still determining factors for future opportunities, with direct repercussions for women’s health [35].

When evaluating the articulation of prenatal care and childbirth care in face of this framework, which is essentially complex due to the quantity of stakeholders, institutions and levels of care which are involved, one can observe that the lack of systemic planning leads regular risk pregnant women to severe maternal condition which are, to a great extent, avoidable.

One limitation of this study, which affects its generalization, is that it was restricted to public facilities. Consequently, it does not represent cases of maternal near miss from private hospitals. Another limitation is that this study does not incorporate some variables that are best addressed by community studies, like cultural aspects, access to health care, and social support, which should be the subject of new studies with qualitative research design, and not just quantitative. Regarding the study design, even though case-control studies may be more prone to bias as compared to cohort studies, they are particularly appropriate for studying rare diseases or outcomes, which is the case of near miss. A cohort study on this subject would require a large number of participants, and even so there would still be a risk of not achieving the needed number of cases. Thus, to minimize the risk of recall and selection bias, two of the most common biases related to case-control studies, we used incident cases and the WHO case definition in the selection of cases.

## 5. Conclusions

The assessment of NM cases constitutes a specific tool for predicting mortality and analyzing maternal health systems. The results of this study can help to strengthen obstetric care through the identification of risk factors for maternal mortality, since women who survive mirror the cases that ended in death.

NM studies were inexistent in the Federal District. They can help subsidize the reformulation of prenatal and childbirth service practices in the public health system. The discovered risk factors point towards the need to prioritize enhancements in work processes, create greater integration between prenatal and childbirth care, lower the number of c-sections, provide adequate care during childbirth, and connect pregnant women and a reference maternity ward since the prenatal stage, avoiding transfers of habitual risk women.

## Figures and Tables

**Table 1:** Distribution of number and percentage of women in potentially life-threatening conditions (PLTC) and near misses (NM) according to sociodemographic characteristics and clinical-obstetric profile in the period between July 2013 and December 2015. Brasilia, Federal District, Brazil.

Variables		PLTC (*n* = 26)		NM (*n* = 148)		*p*-Value ^#^
		*n*	%	*n*	%	
Sociodemographic variables						
Age group (years of age)	<19	0	00	20	13.5	0.062
	19–35	23	88.5	100	67.6	
	>35	3	11.5	28	18.9	
Educational level	Higher education	3	11.5	25	16.9	0.659
	High School	16	61.5	73	49.3	
	Elementary School	7	27.0	48	32.4	
	Illiterate	-	-	2	1.4	
Relationship status	Partner	21	80.8	116	78.4	0.783
	No Partner	5	19.2	32	21.6	
Family income (Minimum Wages)	Up to 2	15	57.7	74	50.0	0.469
	2+	11	42.3	74	50.0	
Perceived ethnicity	White	15	57.7	66	44.6	0.217
	Non-White	11	42.3	82	55.4	
Clinical profile						
BMI Classification	Normal weight	4	15.4	65	43.9	0.051
	Obese	10	38.5	35	23.6	
	Overweight	10	38.5	42	28.4	
	Underweight	2	7.7	6	4.1	
Personal risk antecedents	Yes	12	46.2	65	43.9	
Regular exercise practice	Yes	20	76.9	106	71.6	
Alcohol use *	Yes	3	11.5	12	8.1	
Tobacco Use *	Yes	1	3.8	11	7.4	
Obstetric care						
Prenatal in the Brazil´s unified health system	Yes	23	88.5	114	77.0	0.372
	No prenatal	1	3.8	18	12.2	
Method of service access **	Spontaneous	11	42.3	65	43.9	0.761
	Transfer A	1	3.8	3	2.0	
	Transfer B	5	19.2	24	16.2	
	Transfer C	4	15.4	30	20.3	
	By other service	0	0.0	7	4.7	
	By the same service	5	19.2	19	12.8	
Parity	1	6	23.1	63	42.6	0.163
	>3	7	26.9	33	22.3	
	2 to 3	13	50.0	52	35.1	
Number of prenatal appointments	≥6	17	65.4	79	53.4	0.281
	<6	8	30.8	48	32.4	
	No prenatal	1	3.8	21	14.2	
Prenatal in area of coverage	Yes	19	73.1	89	60.1	0.355
	No prenatal	1	3.8	17	11.5	
Previous c-sections	Yes	11	42.3	36	24.3	
Previous abortion	Yes	7	26.9	45	30.4	
Start of prenatal care (trimester)	1st	22	84.6	104	70.3	0.301
	2nd	1	3.8	16	10.8	
	3rd	3	11.5	28	18.9	
Onset of labor	Spontaneous	6	23.1	34	23.0	0.379
	No labor	15	57.7	63	42.6	
	Induced	0	0.0	15	10.1	
	Abortion	1	3.8	11	7.4	
	Hospitalized before labor	4	15.4	25	16.9	

^#^ Chi-square or Fisher’s exact test. * Use in pregnancy. ** A, transfers made by a rescue/emergency team; B, programmed inter-hospital transfer; and C, non-programmed inter-hospital transfer. BMI: Body Mass Index; Brazil´s unified health system.

**Table 2:** Distribution in the number and percentage of women according to socioeconomic, clinical profile and obstetric antecedents’ variables stratified by cases and controls. Brasilia, Federal District, Brazil.

Variables		Cases (*n =* 174)		Controls (*n =* 358)		*p*-Value ^#^
		*n*	%	*n*	%	
Sociodemographic variables						
Age group (years of age)	<19	20	11.5	43	12.0	0.030
	19–35	123	70.7	280	78.2	
	35+	31	17.8	35	9.8	
Educational level	Higher education	28	16.1	78	21.8	0.271
	High School	89	51.2	190	53.1	
	Elementary School	55	31.6	87	24.3	
	Illiterate	2	1.1	3	0.8	
Relationship status	Partner	137	78.7	203	56.7	<0.001
	No partner	37	21.3	155	43.3	
Family income (Minimum Wages)	Up to 2	89	51.1	233	65.1	<0.001
	2 +	85	48.9	125	34.9	
Perceived ethnicity	White	81	46.6	82	22.9	<0.001
	Non-White	93	53.4	276	77.1	
Clinical profile						
BMI classification	Normal weight	69	39.6	194	54.2	
	Obese	45	25.9	50	14.0	
	Overweight	52	29.9	90	25.1	
	Underweight	8	4.6	24	6.7	0.001
Alcohol use *	Yes	15	8.6	38	10.6	0.471
Regular exercise practice	Yes	126	72.4	50	14.0	<0.001
	No	48	27.6	308	86.0	
Tobacco Use *	Yes	12	6.9	22	6.1	0.740
	No	162	93.1	336	93.9	
Preexisting clinical conditions	Yes	77	44.3	111	31.0	0.003
	No	97	55.7	247	69.0	
Obstetric care						
Prenatal in area of coverage	Yes	108	62.1	216	60.3	
	No	48	27.6	138	38.5	
	No prenatal	18	10.3	4	1.2	<0.001
Previous c-sections	Yes	47	27.0	66	18.4	0.023
	No	127	73.0	292	81.6	
Previous abortions	Yes	52	29.9	75	20.9	0.024
	No	122	70.1	283	79.1	
Prenatal in Brazil´s unified health system	Yes	137	78.8	345	96.4	<0.001
	No	18	10.3	9	2.5	
	No prenatal	19	10.9	4	1.1	
Method of service access **	Spontaneous	76	43.7	214	59.8	<0.001
	Transfer A	4	2.3	58	16.2	
	Transfer B	29	16.7	4	1.1	
	Transfer C	34	19.5	6	1.7	
	For. by other service	7	4.0	3	0.8	
	For. by same service	24	13.8	73	20.4	
Parity	1	69	39.6	155	43.3	0.008
	>3	40	23.0	45	12.6	
	2 to 3	65	37.4	158	44.1	
Number of prenatal assessments	≥6	96	55.2	288	80.4	<0.001
	<6	22	12.6	66	18.4	
	No prenatal	56	32.2	4	1.2	
Start of pre-natal care (trimester)	1st	126	72.4	275	76.8	<0.001
	2nd	17	9.8	67	18.7	
	3rd	31	17.8	12	4.5	
Onset of labor	Spontaneous	40	23.0	138	38.5	<0.001
	No labor	78	44.8	118	33.0	
	Induced	15	8.6	99	27.7	
	Abortion	12	6.9	0	0.0	
	Hospitalized before labor	29	16.7	3	0.8	

^#^ Chi-square or Fisher’s exact test. * Use during pregnancy. ** A, transfers made by a rescue/emergency team; B, programmed inter-hospital transfer; and C, non-programmed inter-hospital transfer. Brazil´s unified health system.

**Table 3:** Results from the simple logistic regression models. Brasília, Federal District, Brazil.

Variables		OR	95% CI	*p*-Value
Sociodemographic variables				
Age group	19–35	1.000	--	--
	<19	1.059	0.598–1.875	0.845
	35 +	2.016	1.189–3.418	0.009
Perceived ethnicity	White	1.000		
	Non-white	2.932	1.991–4.315	<0.001
Education level	Higher Education	1.000	--	--
	High School	1.305	0.792–2.151	0.297
	Elementary School	1.761	1.018–3.047	0.043
	Illiterate	1.857	0.29–11.700	0.510
Relationship status	Partner	1.000	--	--
	No partner	2.827	1.859–4.299	<0.001
Family income (minimum wages)	2+	1.000	--	--
	Up to 2	1.952	1.351–2.821	<0.001
Clinical profile				
Regular exercise practice	Yes	1.000		
	No	2.347	1.501–3.669	<0.001
BMI Classification	Normal Weight	1.000	--	--
	Obesity	2.530	1.554–4.120	<0.001
	Overweight	1.624	1.048–2.518	0.030
	Underweight	0.937	0.402–2.184	0.881
Personal risk antecedents	No	1.000	--	--
	Yes	1.766	1.216–2.567	0.003
Previous SAH	No	1.000	--	--
	Yes	0.254	0.128–0.505	<0.001
Cardiac diseases	No	1.000	--	--
	Yes	0.155	0.041–0.580	0.006
Respiratory diseases	No	1.000	--	--
	Yes	0.260	0.086–0.787	0.017
Kidney diseases	No	1.000	--	--
	Yes	0.078	0.009–0.657	0.019
Falciform Anemia /Thalassemia	No	1.000	--	--
	Yes	0.114	0.032–0.410	0.001
Neurological disease	No	1.000		
	Yes	0.092	0.020–0.425	0.002
Collagenosis	No	1.000	--	--
	Yes	0.160	0.016–1.546	0.113
Obstetric care				
Prenatal in area of coverage.	Yes	1.000	--	--
	No	0.696	0.466–1.039	0.076
	No prenatal	9.000	2.973–27.248	<0.001
Parity	1	1.000	--	--
	2-3	0.924	0.617–1.385	0.702
	>3	1.997	1.197–3.331	0.008
Previous abortions	No	1.000	--	--
	Yes	1.608	1.065–2.429	0.024
Number of previous c-sections	0	1.000	--	--
	≥1	1.637	1.067–2.512	0.024
Number of prenatal appointments	≥ 6	1.000		
	<6	2.507	1.642–3.829	<0.001
	No prenatal	22.000	6.442–75.131	<0.001
Start of prenatal care (trimester)	1st	1.000	--	--
	2nd	0.999	0.622–1.604	0.995
	3rd	0.000	0.000–0.000	0.999
	No prenatal	18.618	4.237–81.812	<0.001
Onset of labor	Spontaneous	1.000	--	--
	No labor	2.281	2.281–1449	<0.001
	Induced	0.523	0.274–0.998	0.049
	Abortion	0.557	0.000 -	0.998
Method of service access *	Spontaneous	1.000	--	--
	Transfer A	0.194	0.068–0.553	0.002
	Transfer B	20.414	6.949–59.973	<0.001
	Transfer C	15.956	6.445–39.501	<0.001
	For. by other service	6.570	1.657–26.053	0.007
	For. by same service	0.926	0.545–1.573	0.775
Pre-natal care done only in public network	Yes	1.000	--	--
	No	5.036	2.209–11.484	<0.001
	No pre-natal	11.962	3.997–35.800	<0.001

* A, transfers made by a rescue/emergency team; B, programmed inter-hospital transfer; and C, non-programmed inter-hospital transfer. SAH: subarachnoid hemorrhage.

**Table 4:** Results from the multiple hierarchical logistic regression model. Brasília, Federal District, Brazil.

		OR	95% CI	*p*-Value
Sociodemographic variables	Non-white	2.973	1.983–4.457	<0.001
	No partner	2.694	1.746–4.161	<0.001
	Up to 2 minimum wages	2.159	1.459–3.196	<0.001
Clinical profile	Obese	20.852	6.958–62.485	<0.001
	Overweight	1.650	1.025–2.657	0.039
	No exercise	2.137	1.280–3.569	0.004
	SAH	0.278	0.120–0.644	0.003
	Heart diseases	0.157	0.038–0.639	0.010
	Kidney disease	0.068	0.007–0.642	0.019
	Falciform Anemia/Thalassemia	0.095	0.025–0.357	<0.001
	Neurological diseases	0.080	0.016–0.397	0.002
Obstetric care	Not getting prenatal care in area of coverage	2.843	1.578–5.124	0.001
	Transfer A *	0.134	0.040–0.447	0.001
	Transfer B *	24.655	6.767–89.828	<0.001
	Transfer C *	11.129	4.088–30.298	<0.001
	Forwarded by same service	6.472	1.343–31.193	0.020
	Forwarded by other service	0.671	0.352–1.279	0.226
	Prenatal not carried out in public network	10.461	3.672–29.805	<0.001
	Less than six prenatal appointments	3.498	1.829–6.690	<0.001
	No labor	2.914	1.634–5.198	<0.001
	Hospitalized before labor	25.205	6.066–104.736	<0.001

* A, transfers made by a rescue/emergency team; B, programmed inter-hospital transfer; and C, non-programmed inter-hospital transfer. SAH: subarachnoid hemorrhage

## Data Availability

All data generated during this study are available within this article.

## References

[1] United Nations (2000). United Nations Millennium Development Goals. Improve Maternal Health.

[2] Dias M.A.B., Domingues R.M.S.M., Schilithz A.O.C., Nakamura-Pereira M., Diniz C.S.G., Brum I.R., Martins A.L., Filha M.M.T., Da Gama S.G.N., Leal M.D.C. (2014). Incidence of maternal near miss in hospital childbirth and postpartum: Data from the Birth in Brazil. Cadernos de Saúde Pública (Rio de Janeiro). Cadernos de Saúde Pública.

[3] Lotufo F.A., Parpinelli M.A., Haddad S.M., Surita F.G., Cecatti J.G. (2012). Applying the new concept of maternal near-miss in an intensive care unit. Clinics.

[4] Drife J.O. (1993). Maternal “near miss” reports?. BMJ.

[5] De Moraes A.P.P., Barreto S.M., Passos V.M.A., Golino P.S., Costa J.E., Vasconcelos M.X. (2013). Severe maternal morbidity: A case-control study in Maranhao, Brazil. Reprod. Health.

[6] Say L., Souza J.P., Pattinson R.C., the WHO working group on Maternal Mortality and Morbidity classifications (2009). Maternal near miss—Towards a standard tool for monitoring quality of maternal health care. Best Pr. Res. Clin. Obstet. Gynaecol..

[7] Pattinson R.C., Hall M. (2003). Near misses: A useful adjunct to maternal death enquiries. Br. Med Bull..

[8] Nakimuli A., Nakubulwa S., Kakaire O., Osinde M.O., Mbalinda S.N., Nabirye R.C., Kakande N., Kaye D.K. (2016). Maternal near misses from two referral hospitals in Uganda: A prospective cohort study on incidence, determinants and prognostic factors. BMC Pregnancy Childbirth.

[9] Souza J., Cecatti J., Parpinelli M., Sousa M., Lago T., Pacagnella R., Camargo R. (2010). Maternal morbidity and near miss in the community: Findings from the 2006 Brazilian demographic health survey. BJOG Int. J. Obstet. Gynaecol..

[10] Souza J.P., Cecatti J.G., Pacagnella R.C., Giavarotti T.M., Parpinelli M.A., Camargo R.S., Sousa M.H. (2010). Development and validation of a questionnaire to identify severe maternal morbidity in epidemiological surveys. Reprod. Health.

[11] Kasahun A.W., Wako W.G. (2018). Predictors of maternal near miss among women admitted in Gurage zone hospitals, South Ethiopia, 2017: A case control study. BMC Pregnancy Childbirth.

[12] Goffman D., Madden R.C., Harrison E.A., Merkatz I.R., Chazotte C. (2007). Predictors of maternal mortality and near-miss maternal morbidity. J. Perinatol..

[13] Hosmer D.W., Lemeshow S. (2004). Applied Logistic Regression.

[14] Victora C.G., Huttly S.R., Fuchs S.C., Olinto M.T. (1997). The role of conceptual frameworks in epidemiological analysis: A hierarchical approach. Int. J. Epidemiol..

[15] Wold Health Organization (2009). Women and Health: Evidence for Today, Tomorrow’s Schedule. http://www.who.int/ageing/mulheres_saude.pdf.

[16] Waterstone M., Bewley S., Wolfe C., Murphy J.D. (2001). Incidence and predictors of severe obstetric morbidity: Case-control study. BMJ.

[17] Tangel V., White R.S., Nachamie A.S., Pick J.S. (2019). Racial and Ethnic Disparities in Maternal Outcomes and the Disadvantage of Peripartum Black Women: A Multistate Analysis, 2007–2014. Am. J. Perinatol..

[18] Creanga A.A., Bateman B.T., Kuklina E.V., Callaghan W.M. (2014). Racial and ethnic disparities in severe maternal morbidity: A multistate analysis, 2008–2010. Am. J. Obstet. Gynecol..

[19] Tunçalp Ö., Souza J., Hindin M., Santos C., Oliveira T., Vogel J., Togoobaatar G., Ha D., Say L., Gülmezoglu A. (2014). Education and severe maternal outcomes in developing countries: A multicountry cross-sectional survey. BJOG Int. J. Obstet. Gynaecol..

[20] Amaral E., Souza J.P., Surita F., Luz A.G., Sousa M.H., Cecatti J.G., Campbell O. (2011). A population-based surveillance study on severe acute maternal morbidity (near-miss) and adverse perinatal outcomes in Campinas, Brazil: The Vigimoma Project. BMC Pregnancy Childbirth.

[21] Oliveira F.C., Surita F.G., Silva J.L.P.E., Cecatti J.G., Parpinelli M.A., Haddad S.M., Costa M.L., Pacagnella R.C., Sousa M.H., The Brazilian Network for Surveillance of Severe Maternal Morbidity Study Group (2014). Severe maternal morbidity and maternal near miss in the extremes of reproductive age: Results from a national cross- sectional multicenter study. BMC Pregnancy Childbirth.

[22] Giordano J.C., Parpinelli M.A., Cecatti J.G., Haddad S.M., Costa M.L., Surita F.G., Silva J.L.P.E., Sousa M.H. (2014). The Burden of Eclampsia: Results from a Multicenter Study on Surveillance of Severe Maternal Morbidity in Brazil. PLoS ONE.

[23] Luexay P., Malinee L., Pisake L., Marie-Hélène B.-C. (2014). Maternal near-miss and mortality in Sayaboury Province, Lao PDR. BMC Public Health.

[24] Assarag B., Dujardin B., Delamou A., Meski F.-Z., De Brouwere V. (2015). Determinants of Maternal Near-Miss in Morocco: Too Late, Too Far, Too Sloppy?. PLoS ONE.

[25] Zanette E., Parpinelli M.A., Surita F.G., Costa M.L., Haddad S.M., Sousa M.H., Silva J.L.P.E., Souza J.P., Cecatti J.G., The Brazilian Network for Surveillance of Severe Maternal Morbidity Group (2014). Maternal near miss and death among women with severe hypertensive disorders: A Brazilian multicenter surveillance study. Reprod. Health.

[26] Pacheco A.J.C., Katz L., Souza A.S.R., De Amorim M.M.R. (2014). Factors associated with severe maternal morbidity and near miss in the São Francisco Valley, Brazil: A retrospective, cohort study. BMC Pregnancy Childbirth.

[27] Knight M., Lewis G., Acosta C.D., Kurinczuk J.J. (2014). Maternal near-miss case reviews: The UK approach. BJOG Int. J. Obstet. Gynaecol..

[28] Hinton L., Locock L., Knight M. (2014). Experiences of the quality of care of women with near-miss maternal morbidities in the UK. BJOG: Int. J. Obstet. Gynaecol..

[29] Haddad S.M., Cecatti J.G., Souza J.P., Sousa M.H., Parpinelli M.A., Costa M.L., Pacagnella R.C., Brum I.R., Filho O.B.M., Feitosa F.E. (2014). Applying the Maternal Near Miss Approach for the Evaluation of Quality of Obstetric Care: A Worked Example from a Multicenter Surveillance Study. BioMed Res. Int..

[30] Zwart J., Richters J., Ory F., Vries J.I.P.D., Bloemenkamp K., Van Roosmalen J. (2008). Severe maternal morbidity during pregnancy, delivery and puerperium in the Netherlands: A nationwide population-based study of 371 000 pregnancies. BJOG Int. J. Obstet. Gynaecol..

[31] Donati S., Senatore S., Ronconi A., Basevi V., Casotto V., Cernigliaro A., Dardanoni G., De Nisi M., Di Lallo D., Dubini V. (2012). Obstetric near-miss cases among women admitted to intensive care units in Italy. Acta Obstet. Gynecol. Scand..

[32] Litorp H., Kidanto H.L., Rööst M., Abeid M., Nyström L., Essén B. (2014). Maternal near-miss and death and their association with caesarean section complications: A cross-sectional study at a university hospital and a regional hospital in Tanzania. BMC Pregnancy Childbirth.

[33] Mantel G.D., Buchmann E., Rees H., Pattinson R.C. (1998). Severe acute maternal morbidity: A pilot study of a definition for a near-miss. BJOG Int. J. Obstet. Gynaecol..

[34] Geller S.E., Rosenberg D., Cox S., Brown M., Simonson L., Kilpatrick S. (2004). A scoring system identified near-miss maternal morbidity during pregnancy. J. Clin. Epidemiol..

[35] Rosendo T.M.S.S., Roncalli A.G. (2016). Maternal near misses and health inequalities: An analysis of contextual determinants in the State of Rio Grande do Norte, Brazil. Brasil. Ciência Saúde Coletiva.

